# Paricalcitol-mediated vitamin D receptor activation attenuates neuronal ferroptosis via cAMP-PKA-DRP1 signaling pathway after intracerebral hemorrhage

**DOI:** 10.1016/j.neurot.2025.e00767

**Published:** 2025-10-21

**Authors:** Mi Tian, Xin Li, Dongqing Qi, Pengju Wei, Peng Jin

**Affiliations:** a Department of Intensive Care Unit, The First Affiliated Hospital of USTC, Division of Life Sciences and Medicine, University of Science and Technology of China, Hefei, Anhui, China; b Department of Intensive Care Unit, Huashan Hospital, Fudan University, Shanghai, China; c Department of Anesthesiology, Shanghai Chest Hospital, Shanghai Jiao Tong University, School of Medicine, Shanghai, China; d Department of Rehabilitation Medicine, The First Affiliated Hospital of USTC, Division of Life Sciences and Medicine, University of Science and Technology of China, Hefei, Anhui, China; e Institute of Biomedicine and Biotechnology, Shenzhen Institute of Advanced Technology, Chinese Academy of Sciences, Shenzhen, Guangdong, China

**Keywords:** Intracerebral hemorrhage, Vitamin D receptor, Ferroptosis, Mitochondrial fission, Paricalcitol

## Abstract

The Vitamin D Receptor (VDR) is an emerging therapeutic target for neurological injuries, yet its role in neuronal ferroptosis and mitochondrial dynamics following intracerebral hemorrhage (ICH) remains undefined. This study aimed to determine if VDR activation protects neurons by regulating mitochondrial fission via the Cyclic Adenosine Monophosphate - Protein Kinase A - Dynamin-related Protein 1 (cAMP-PKA-DRP1) signaling pathway. We utilized a mouse ICH model and a hemin-induced injury model in primary neurons to evaluate the neuroprotective efficacy of the selective VDR agonist, Paricalcitol (PAL). Our results first establish that VDR is a key neuronal target, as its expression is robustly upregulated in perihematomal neurons both in vivo and in vitro. Systemic PAL administration in mice conferred significant neuroprotection, reducing acute neuronal death, suppressing ferroptosis, and preventing excessive mitochondrial fission, which translated into lasting improvements in long-term cognitive function and synaptic integrity. Mechanistically, we demonstrate that PAL's anti-ferroptotic action is a direct neuroprotective effect, independent of microglial hematoma clearance. The core signaling cascade involves VDR-dependent activation of the cAMP-PKA pathway, leading to an increase in the inhibitory phosphorylation of DRP1 at Ser637. The necessity of this pathway was confirmed as the protective effects of PAL were abrogated by VDR knockdown or cAMP inhibition. Critically, its sufficiency was demonstrated as direct activation of the pathway with an agonist mimicked PAL's anti-ferroptotic effects. Collectively, these findings reveal that VDR activation by paricalcitol ameliorates neuronal injury after ICH by directly inhibiting ferroptosis through the cAMP-PKA-DRP1-mediated preservation of mitochondrial integrity, highlighting a potent therapeutic strategy.

## Graphical abstract

**VDR modulates ferroptosis following experimental ICH via the cAMP/PKA/DRP1 pathway.** Following ICH, VDR expression increases and localizes to neurons. Activation of VDR with paricalcitol (PAL) reduces mitochondrial dysfunction, inhibits ferroptosis markers (4-HNE, ACSL4, MDA), and enhances phosphorylation of DRP1 at Ser637 via cAMP-PKA signaling, thereby preserving mitochondrial integrity and reducing neuronal death. These effects improve both short-term and long-term neurobehavioral outcomes in ICH models.

**Figure:**
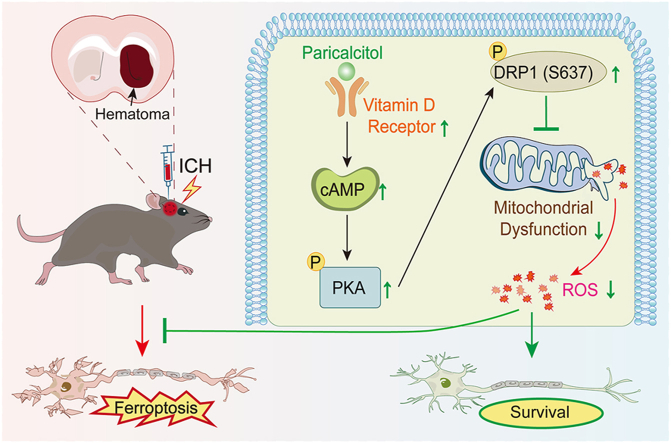

## Introduction

Intracerebral hemorrhage (ICH) is a severe cerebrovascular condition associated with significant mortality and disability rates [1]. Following ICH, blood infiltrates the brain tissue, releasing toxic substances such as iron ions and heme, which trigger secondary damage, including oxidative stress, inflammatory responses, and neuronal death [2]. Among the different types of cell death, ferroptosis—a regulated process characterized by iron accumulation and lipid peroxidation—has attracted considerable attention in recent years [3]. Ferroptosis is characterized by iron metabolism dysregulation and mitochondrial dysfunction, with excessive mitochondrial fission being a key trigger of this process [4,5]. Therefore, inhibiting mitochondrial fission and preserving mitochondrial function could serve as an effective strategy to alleviate ferroptosis, reducing neuronal damage following ICH.

The Vitamin D receptor (VDR) is a nuclear receptor that has garnered increasing attention for its neuroprotective role [6]. Upon activation, VDR has been shown to exert anti-inflammatory, antioxidant, and anti-apoptotic effects in various neurodegenerative diseases, such as Alzheimer's and Parkinson's diseases [7,8]. Moreover, in non-neurological diseases like diabetic nephropathy and cardiovascular diseases, VDR has been demonstrated to protect cells by regulating mitophagy, reducing mitochondrial fission, and maintaining mitochondrial function [[9], [10], [11]]. Specifically within the context of ICH, VDR activation is also known to be beneficial by promoting the differentiation of reparative macrophages, which enhances hematoma clearance and improves neurological recovery [12]. However, whether VDR can modulate mitochondrial fission to inhibit neuronal ferroptosis and thereby exert neuroprotective effects in the context of ICH remains unexplored.

Dynamin-related protein 1 (DRP1) is a key regulator of mitochondrial fission [13]. During the mitochondrial fission process, DRP1 is recruited to the outer mitochondrial membrane, where it interacts with other proteins to induce mitochondrial division [14,15]. Under pathological conditions, DRP1 overactivation leads to excessive mitochondrial fission, leading to mitochondrial dysfunction and elevated production of reactive oxygen species (ROS), decreased Adenosine triphosphate (ATP) synthesis, and ultimately, ferroptosis [16,17]. DRP1 activity is regulated by its phosphorylation status, particularly at the Ser637 site, where phosphorylation inhibits its activity and reduces mitochondrial fission [18]. Studies have demonstrated that VDR activation can modulate mitochondrial dynamics by reducing DRP1 expression and increasing phosphorylation at the Ser637 site, thereby inhibiting mitochondrial fission and preserving mitochondrial function [19,20]. Nevertheless, the exact molecular mechanisms governing this regulation remain unclear, especially in the context of ICH.

There is evidence suggesting that VDR modulates various physiological functions through the cAMP-PKA (Protein Kinase A) signaling pathway in other disease models and cell systems [21,22]. VDR activation increases intracellular Cyclic adenosine monophosphate (cAMP) levels, which subsequently activate PKA [23]. It has been demonstrated that PKA can phosphorylate DRP1 at Ser637, inhibiting its activity and reducing mitochondrial fission [24]. Therefore, it is hypothesized that VDR may modulate DRP1 phosphorylation via the cAMP-PKA signaling pathway to exert mitochondrial protective effects under certain pathological conditions. However, whether VDR regulates DRP1 and mitochondrial fission through this pathway in ICH has not yet been thoroughly investigated.

Herein, we propose VDR as a key regulator of mitochondrial dynamics and ferroptosis in ICH. VDR activation would suppress DRP1-mediated mitochondrial fission and protect against neuronal ferroptosis via the cAMP-PKA signaling pathway. The modulation of VDR could mitigate mitochondrial dysfunction and reduce neuronal injury following ICH.

## Material and methods

### Experiment design

This study aimed to explore the role of VDR activation in reducing neuronal injury and ferroptosis following ICH through its effects on mitochondrial dynamics. Five experimental sub-groups were established, and animals and cell models were randomly allocated to these groups. To minimize bias, researchers conducting surgeries, outcome evaluations, and data analyses were blinded to group assignments.

#### Experiment 1 protein expression and cellular localization of VDR after ICH

Western blot analysis was performed to examine the temporal expression level of VDR in a mouse ICH model at sham group and different timepoints after ICH (6 ​h, 12 ​h, 24 ​h, 72 ​h, and 7 ​d). Concurrently, an in vitro neuronal ICH model was employed to assess VDR expression at control group and different timepoints after hemin stimulation (6 ​h, 12 ​h, 24 ​h, and 48 ​h). To determine VDR localization within neurons, double immunofluorescence staining for VDR and the neuronal marker NeuN was conducted at 24 ​h in both vivo and vitro models.

#### Experiment 2: paricalcitol attenuates acute brain injury, neuronal death, and ferroptosis *in vivo*

To evaluate the in vivo therapeutic efficacy of the VDR agonist paricalcitol (PAL) and to investigate its underlying mechanisms following ICH, we first conducted a dose-response study. Mice were randomly divided into groups: Sham, ICH ​+ ​Vehicle, and ICH ​+ ​PAL (at 0.1, 0.5, or 1.0 ​μg/kg). Neurological function was assessed using the Modified Garcia, corner turn, and forelimb placement tests at 1 day, 3 days and 7 days post-ICH to determine the optimal therapeutic dose.

Based on these outcomes, the optimal dose of PAL was used for all subsequent mechanistic studies. A separate cohort of mice was randomized into three groups (Sham, ICH ​+ ​Vehicle, ICH ​+ ​PAL) to assess pathological changes at 24 ​h post-ICH. In these animals, we evaluated [1]: Neuronal Death, using Fluoro-Jade C (FJC) and TUNEL staining [2]; Ferroptosis, by performing immunofluorescence for the lipid peroxidation marker 4-HNE and by measuring brain tissue levels of MDA and GSH via ELISA; and [3] Mitochondrial Damage, by assessing ultrastructural changes in mitochondria with transmission electron microscopy (TEM).

#### Experiment 3 isolating the direct, neuron-intrinsic anti-ferroptotic effects of paricalcitol

This experiment was designed to test the alternative hypothesis that the neuroprotective effects of PAL are an indirect consequence of enhanced phagocytic activity. To achieve this, we used clodronate liposomes to deplete phagocytic microglia/macrophages in the ICH model. Mice were randomized into four experimental groups [1]: ICH ​+ ​Control Liposomes ​+ ​Vehicle [2]; ICH ​+ ​Control Liposomes ​+ ​PAL [3]; ICH ​+ ​Clodronate Liposomes ​+ ​Vehicle; and [4] ICH ​+ ​Clodronate Liposomes ​+ ​PAL. The efficiency of microglial/macrophage depletion was confirmed using Iba-1 immunofluorescence. The primary outcome—the direct anti-ferroptotic efficacy of PAL—was then evaluated by collecting brain tissue at 24 ​h post-ICH and performing Western blot analysis for the key ferroptosis markers 4-HNE and ACSL4.

#### Experiment 3 long-term cognitive and memory effects of paricalcitol treatment

To investigate the long-term effects of PAL after ICH, 30 mice were randomly assigned to three groups: sham, ICH ​+ ​vehicle, and ICH ​+ ​PAL (0.5 ​μg/kg) (n ​= ​10 per group). PAL was administered daily through intraperitoneal injection from days 1–7 post-surgery. Cognitive function was assessed using the Morris water maze test from days 23–28 post-ICH. After the behavioral tests, the mice were euthanized, and Nissl staining and Golgi-Cox Staining were conducted to examine neuronal degeneration.

#### Experiment 5 exploring the molecular mechanisms of VDR activation via Cyclic Adenosine Monophosphate - Protein Kinase A - Dynamin-related Protein 1 (cAMP-PKA-DRP1) signaling pathway

To establish VDR-dependency, an in vivo siRNA-mediated knockdown study was performed. Mice were randomly assigned to four groups [1]: ICH ​+ ​Scrambled (Scr) siRNA ​+ ​Vehicle [2]; ICH ​+ ​Scr siRNA ​+ ​PAL [3]; ICH ​+ ​VDR siRNA ​+ ​Vehicle; and [4] ICH ​+ ​VDR siRNA ​+ ​PAL. VDR-targeting siRNA or Scr siRNA was administered via intracerebroventricular injection 48 ​h prior to ICH induction. PAL or vehicle was administered as previously described. At 72 ​h post-ICH, neurological function was assessed. Brain tissues were then harvested for Western blot analysis to determine the knockdown efficiency of VDR and to quantify the levels of the ferroptosis markers ACSL4 and 4HNE.

To investigate the downstream signaling pathway, a pharmacological inhibition study was conducted using the adenylyl cyclase inhibitor SQ22536. Mice were randomized into five groups [1]: Sham [3]; ICH ​+ ​Vehicle [3]; ICH ​+ ​PAL [4]; ICH ​+ ​PAL ​+ ​DMSO (vehicle for inhibitor); and [5] ICH ​+ ​PAL ​+ ​SQ22536. Following ICH induction and PAL treatment, SQ22536 was administered via intraperitoneal injection. At 72 ​h, neurological function was assessed. Brain tissues were then collected for Western blot analysis of key pathway proteins (p-PKA, p-Drp1 Ser637) and ferroptosis markers (ACSL4, 4HNE), and for ELISA-based quantification of MDA and GSH levels.

#### Experiment 6 in vitro Validation of Paricalcitol's neuron-intrinsic protective mechanism

To confirm a direct neuroprotective mechanism, we used an in vitro model of primary cortical neurons subjected to hemin-induced hemorrhagic stress. The primary efficacy of paricalcitol (PAL) was evaluated by quantifying its effect on hemin-induced apoptosis using Annexin V/PI flow cytometry. To investigate the underlying mechanism, we assessed intracellular reactive oxygen species (ROS) and mitochondrial membrane potential (ΔΨm). Mitochondrial health was evaluated using two complementary methods: flow cytometry with JC-1 staining and in situ fluorescence microscopy of JC-1 on intact, adherent neurons.

The dependency of this protective action on the proposed signaling pathway was then investigated through two interventional experiments. To test the role of VDR, neurons were first transfected with VDR-targeting siRNA prior to hemin and PAL treatment. To confirm the involvement of the downstream cascade, neurons were co-treated with the adenylyl cyclase inhibitor SQ22536. In both sets of experiments, the final extent of neuroprotection was determined by quantifying apoptosis via flow cytometry.

### Animals

Male C57BL/6 mice (8–10 weeks old, weighing 22–25 ​g) were purchased from Jicui Yaokang Biotechnology Co., Ltd. (Jiangsu, China). The mice were maintained under controlled environmental conditions, including a 12-h light/dark cycle, a temperature of 22 ​± ​2 ​°C, and 50–60 ​% humidity, with unrestricted access to food and water. All animal experiments were approved by the Institutional Animal Care and Use Committee (IACUC) of the First Affiliated Hospital of the University of Science and Technology of China (2022-N(A)-300) and performed in accordance with ethical standards. Efforts were made to follow the 3Rs principles (Replacement, Reduction, and Refinement) to minimize animal suffering and reduce the number of animals used. Postoperative care and monitoring were conducted to ensure animal well-being.

### Animal ICH model

ICH model was generated by autologous blood injection technique [25]. Mice were anesthetized with ketamine and xylazine and secured in a stereotaxic frame. A burr hole was drilled at coordinates 0.5 ​mm anterior-posterior, 2.0 ​mm lateral, and 3.5 ​mm depth relative to the bregma. A total of 30 ​μL of blood, collected from the femoral artery, was slowly injected into the right basal ganglia over 5 ​min. To prevent backflow, the needle remained in place for 5 ​min before being carefully withdrawn. The burr hole was sealed, the incision sutured, and the mice were given post-surgical care and monitoring.

### Primary neuron culture and ICH model *in vitro*

Primary cortical neurons were isolated from fetal (E16–18) C57BL/6J mice (SLAC, Shanghai, China) and cultured according to standard protocols [26]. In brief, under deep anesthesia, fetal mice were decapitated and sterilized with 75 ​% ethanol. The cerebral cortices were carefully dissected, and the leptomeninges, blood vessels, and white matter were removed under a microscope in HBSS (Thermo Fisher Scientific, USA, Cat# 14065056). The tissue was digested with 0.125 ​% trypsin at 37 ​°C for 5 ​min, followed by filtration through a 40 ​μm strainer and centrifugation at 1500 ​rpm for 5 ​min. The resulting cell pellets were resuspended in Dulbecco's Modified Eagle Medium (Thermo Fisher Scientific, USA, Cat# 30030) supplemented with FBS and penicillin-streptomycin and plated on poly-d-lysine-coated dishes. After 2 ​h, the medium was replaced with Neurobasal Medium (Thermo Fisher Scientific, USA, Cat#21103049) containing GlutaMAX-I, B27 supplement, and penicillin-streptomycin. Cultures were maintained at 37 ​°C in a humidified incubator with 5 ​% CO_2_, with medium changes every 2 days.

For the in vitro ICH model, neurons were treated with 2 ​μM Hemin (Millipore Sigma, USA, Cat#H9039) dissolved in culture medium for 24 ​h to mimic ICH conditions, as described previously [27]. All experiments were conducted at least three times to ensure reproducibility.

### Drug administration

#### Paricalcitol administration

Paricalcitol (Sigma-Aldrich, USA, Cat#HY-50919) was dissolved in sterile saline and administered via intraperitoneal injection (i.p.) at doses of 0.1 ​μg/kg, 0.5 ​μg/kg, and 1.0 ​μg/kg. The initial dose was given 1 ​h after ICH induction. For evaluating long-term behavioral outcomes, daily injections were continued for 7 consecutive days post-ICH. These doses were selected based on preliminary studies identifying effective neuroprotective concentrations for modulating VDR signaling pathways [28,29].

#### Administration of cAMP inhibitor SQ22536

To investigate the role of the cAMP-PKA-DRP1 signaling pathway, the cAMP inhibitor SQ22536 (Tocris Bioscience, USA, Cat#1435/10) was administered intracerebroventricularly (ICV) at a dose of 2 ​nmol per mouse [30]. SQ22536 was dissolved in artificial cerebrospinal fluid (aCSF) and injected 1 ​h prior to ICH induction. The injection was performed using a Hamilton syringe, targeting the lateral ventricle under stereotaxic guidance (coordinates: 0.2 ​mm posterior, 1.0 ​mm lateral to bregma, and 2.5 ​mm deep).

#### VDR siRNA administration

For VDR silencing in vivo, VDR-targeting siRNA (Sigma-Aldrich, USA) was administered via ICV injection using the same procedure and stereotaxic coordinates as SQ22536. A single dose of VDR siRNA or scrambled control siRNA was delivered 24 ​h before ICH induction. VDR knockdown efficiency was validated by Western blot analysis. In vitro experiments, VDR siRNA was transfected into primary cortical neurons using a liposome-based transfection reagent (Thermo Fisher Scientific, USA). The siRNA-transfection reagent complexes were prepared according to the manufacturer's instructions and applied at a final concentration of 20–50 ​nM. Knockdown efficiency was confirmed by Western blot analysis 48 ​h after transfection.

### Behavioral tests

Based on previous studies, we employed a series of behavioral tests, including the Garcia test, corner turn test, forelimb placement test, and Morris Water Maze, to comprehensively assess neurological and cognitive functions in mice [25]. All behavioral assessments in this study were conducted using a double-blind method. The Garcia test evaluated six parameters—spontaneous activity, limb symmetry, climbing, forelimb extension, body proprioception, and response to vibrissae stimulation—each scored from 0 to 3, with a maximum score of 18 indicating better function. The corner turn test measured motor asymmetry by recording the turning direction (left or right) of mice placed between two angled boards (30°). Ten trials were conducted, and the percentage of ipsilateral turns, reflecting impairment, was calculated. The forelimb placement test assessed sensorimotor function by recording the number of successful forelimb placements onto a table edge after whisker stimulation, with 10 trials performed per side. Cognitive function was evaluated on day 28 post-ICH using the Morris Water Maze, Mice underwent a five-day training period to locate a hidden platform in a pool filled with opaque water, with the time taken to find the platform (latency) recorded, and memory retention was assessed on day 6 by measuring the time spent in the target quadrant after the platform was removed.

### TUNEL staining

Terminal deoxynucleotidyl transferase dUTP nick-end labeling (TUNEL) was performed using the Apoptosis Detection Kit (Roche, USA, Cat#11684795910) to assess neuronal apoptosis in the peri-hematoma region 24 ​h post-ICH, following the manufacturer's instructions. TUNEL-positive neurons were manually counted under ​× ​200 magnification, and the results were expressed as the percentage of TUNEL-positive neurons relative to the total neuronal population.

### FJC staining

Fluoro-Jade C (FJC) staining was performed to assess neurodegeneration in brain sections collected 24 ​h after ICH. The sections were fixed with 4 ​% paraformaldehyde, mounted on slides, and rehydrated before incubation in FJC solution, following the manufacturer's instructions (Millipore Sigma, USA, Cat#AG325). After washing and air-drying, the sections were counterstained with DAPI to visualize nuclei. FJC-positive neurons in the perihematomal region were observed using fluorescence microscopy, and neuronal damage was quantified by counting the number of FJC-positive neurons per square millimeter.

### Elisa assay

ELISA was performed to evaluate ferroptosis markers in the ipsilateral brain hemisphere [31]. After PBS perfusion, brain tissue was collected and homogenized in chilled lysis buffer. The supernatant was isolated via centrifugation at 4 ​°C and 14,000 ​rpm for 30 ​min. MDA levels were determined using the MDA Assay Kit (Beyotime, China, Cat#S0131S) via a colorimetric reaction with thiobarbituric acid at 532 ​nm. GSH levels were measured using the GSH Assay Kit (Beyotime, China, Cat#S0053) through a reaction involving glutathione reductase, with absorbance read at 412 ​nm. Concentrations were calculated using respective formulas.

### Electron microscopy for mitochondrial damage

Under deep anesthesia with 5 ​% isoflurane, mice were perfused with a fixative solution containing 4 ​% paraformaldehyde and 2.5 ​% glutaraldehyde. Brain tissue was dissected into 1 ​mm^3^ cubes and initially fixed in 2.5 ​% glutaraldehyde, followed by secondary fixation with 1 ​% osmium tetroxide. The samples were dehydrated using a graded ethanol series (50 ​%, 70 ​%, 90 ​%, and 100 ​%), then embedded and sectioned for further analysis. Sections were stained with 3 ​% uranyl acetate and lead citrate and examined using a transmission electron microscope (TEM, Hitachi H 7500, Japan). Impaired mitochondria were identified at 3000 ​× ​magnification based on features such as atrophy, cristae loss, and increased membrane density. For quantitative analysis, TEM images were processed using ImageJ. In neuronal somas, mitochondrial contours were manually traced to measure cross-sectional areas (μm^2^) and aspect ratios (major axis/minor axis). Axonal quantification was not performed due to insufficient clear cross-sections in the captured images.

### Golgi-Cox Staining

At 28 days post-ICH, brains were harvested and immersed in Golgi-Cox solution (1 ​% potassium dichromate, 1 ​% mercuric chloride, 0.8 ​% potassium chromate) for 14 days in the dark, then transferred to 30 ​% sucrose for protection [32]. We cut 150-μm coronal sections on a vibratome, developed them in 28 ​% ammonium hydroxide for 30 ​min, fixed in photographic fixer, and mounted/dehydrated the slides. Dendritic spines on CA1 pyramidal neurons were viewed with a bright-field microscope (Olympus BX51) at 1000×. Neurolucida software (MBF Bioscience) helped quantify spine density on apical segments (50–150 ​μm from soma) from 10 to 15 neurons per animal (n ​= ​6/group).

### Mitochondrial membrane potential measurement

Mitochondrial membrane potential (ΔΨm) was measured using the MitoProbe™ JC-1 Assay Kit (Thermo Fisher, USA). At the end of the designated treatment, the culture medium was removed, and cells were digested and centrifuged at 1000 ​× ​*g* for 5 ​min in 1.5 ​mL tubes. The cell pellet was resuspended in DMEM containing the JC-1 dye and incubated for 20 ​min. After staining, cells were centrifuged again at 1000 ​× ​*g* for 5 ​min, washed twice with PBS, and resuspended in PBS. Fluorescence intensity was analyzed using a flow cytometer (BD Biosciences, USA), and the results were processed with FlowJo software. Within FlowJo, a compensation matrix was first established using single-stain controls to correct for spectral overlap between FITC and PE channels. A sequential gating strategy was then employed: debris was first excluded based on Forward and Side Scatter (FSC-A vs. SSC-A), followed by doublet discrimination (FSC-A vs. FSC-H) to isolate the single-cell population. On this population, analytical gates for healthy (PE-high) and depolarized (FITC-high) cells were set based on two parallel controls: unstained cells (blank control) were used to define baseline fluorescence, while cells treated with 50 ​μM CCCP (positive control) were used to define the maximally depolarized population.

### Intracellular ROS measurement

This method was used to assess intracellular ROS levels in the cell model. ROS detection was performed using DCFH-DA staining (Applygen, China). Harvested cells were incubated with 2.5 ​μM DCFH-DA for 30 ​min, followed by two PBS washes. Approximately 1 ​× ​10^5^ ​cells per sample, as recommended by the manufacturer, were analyzed for ROS levels using a flow cytometer (BD Biosciences, USA). Results were processed using FlowJo software.

### Annexin V and PI staining

Annexin V and PI staining was used to detect apoptotic neurons in vitro, following the protocol described in previous study [26] and the manufacturer's instructions for the FITC Annexin V Apoptosis Detection Kit (BD Biosciences, USA, Cat#556547). Stained cells were analyzed using a flow cytometer (BD Biosciences, USA). Neurons negative for both FITC and PI were classified as surviving, while early apoptotic neurons were identified as FITC+/PI-.

### Immunofluorescence staining

Double-immunofluorescence staining was conducted following established protocols [33]. Mice were intracardially perfused with PBS followed by formalin, and brain tissues were fixed in formalin for 24 ​h before dehydration in sucrose solutions. The tissues were embedded in OCT, frozen, and sectioned into 10-μm coronal slices using a cryostat (Leica Microsystems, Germany). Sections were washed three times in 0.01 ​M PBS (10 ​min each) and blocked with 5 ​% donkey serum at room temperature for 2 ​h. Primary antibodies, including anti-VDR (1:50, Abcam, USA, Cat#ab3508), anti-4HNE (1:50, Thermo Fisher, USA, Cat#MA5-27570), and anti-NeuN (1:100, Abcam, USA, Cat#ab177487), were applied overnight at 4 ​°C. The following day, sections were incubated with fluorescence-conjugated secondary antibodies (1:500, Beyotime, China) for 1 ​h at room temperature. Staining was examined under a fluorescence microscope (Leica Microsystems, Germany). To ensure specificity, isotype control (rabbit IgG, 1:50) and secondary antibody-only sections were processed in parallel, confirming absence of non-specific staining. Staining was examined under a fluorescence microscope.

### Western blot

Western blotting was conducted as previously described [34]. Mice were anesthetized with isoflurane and perfused with ice-cold PBS. Ipsilateral brain hemispheres were collected, flash-frozen in liquid nitrogen, and stored at −80 ​°C. Tissues were homogenized in RIPA lysis buffer (Beyotime, China, Cat#P0038) with protease inhibitors, incubated for 15 ​min, and centrifuged at 14,000 ​× ​*g* at 4 ​°C for 30 ​min. The supernatant was collected, and protein concentration was determined using the DC Protein Assay (Beyotime, China, Cat#P0011). Equal amounts of protein were separated via SDS-PAGE, transferred to nitrocellulose membranes, and blocked with 5 ​% nonfat milk for 2 ​h at room temperature. Membranes were incubated overnight at 4 ​°C with primary antibodies, including anti-VDR (1:1000, Abcam, USA, Cat#ab3508), anti-cAMP (1:2000, Cell Signaling Technology, USA, Cat#35031), anti-PKA (1:2000, Thermo Fisher, USA, Cat#PA5-17626), anti-p-PKA (1:500, Thermo Fisher, USA, Cat#711615), anti-p-DRP1 (Ser637) (1:500, Thermo Fisher, USA, Cat# PA5-37534), anti-4HNE (1:500, Thermo Fisher, USA, Cat#MA5-27570), anti-ACSL4 (1:1000, Abcam, USA, Cat#ab155282) and anti-β-actin (1:3000, Cell Signaling Technology, USA, Cat#4967). The next day, membranes were treated with secondary antibodies (1:3000, Beyotime, China) at room temperature, and bands were visualized using the ECL Plus chemiluminescence kit (Beyotime, China, Cat#P0018S). Band intensity was quantified using ImageJ software (NIH, USA), with β-actin serving as an internal control for normalization.

### Statistical analysis

Data are presented as mean ​± ​SD. Statistical analysis was performed using Prism 10.0 (GraphPad, CA, USA) and SPSS 23.0 (IBM, NY, USA). Normality was assessed with the Kruskal-Wallis test, and group differences were analyzed using one-way ANOVA with Tukey's post hoc test. A p-value <0.05 was deemed significant.

## Results

### Animal use and mortality

A total of 260 male C57BL/6 mice were used in this study. Among these, 48 mice were assigned to the Sham group, with no mortality observed throughout the experimental period. 188 mice were subjected to the operation group, of which 9 mice did not survive the procedure or the immediate postoperative period, resulting in a mortality rate of 3.46 ​%. All surviving mice were included in subsequent analyses (Table S1).

### Time-course and cellular localization of VDR after ICH

**Fig. 1:**
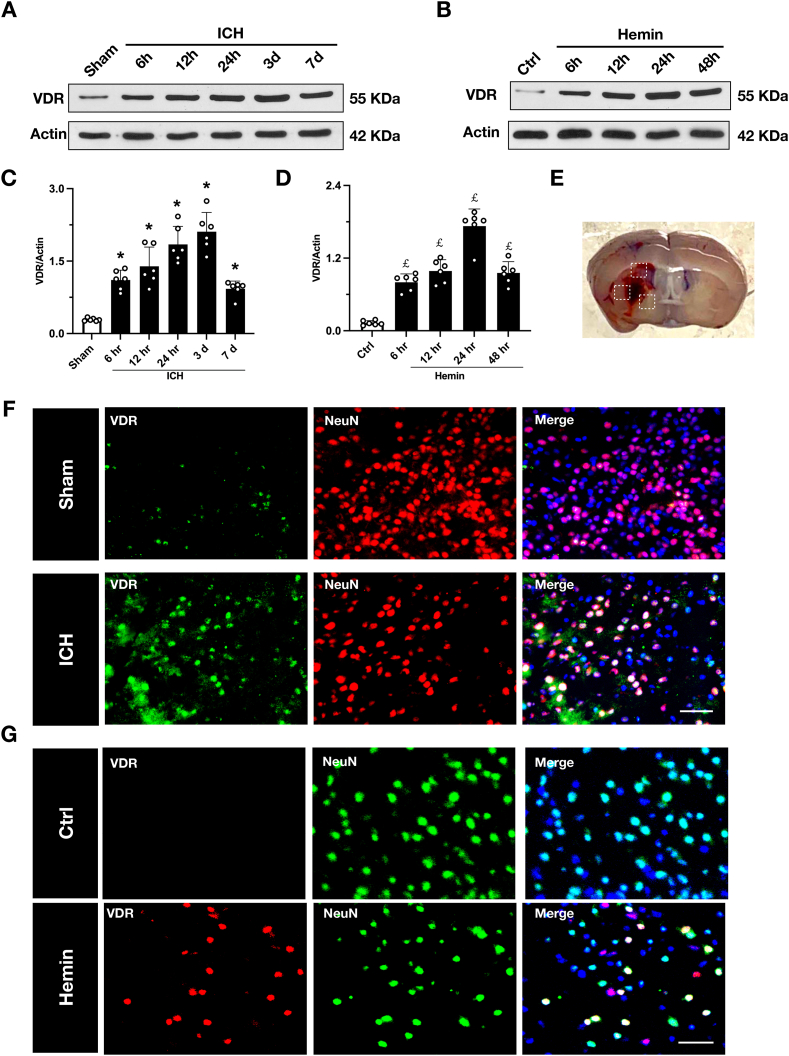
**VDR is a neuronal target upregulated after ICH injury in vivo and in vitro.** (A, C) Representative immunoblot bands and quantitative analysis of VDR protein levels in the sham group and at 6 ​h, 12 ​h, 1 ​d, 3 ​d, and 7 ​d post-ICH (n ​= ​6/group). (B, D) Representative immunoblot bands and quantitative analysis of VDR in the control group and at 6 ​h, 12 ​h, 24 ​h, and 48 ​h after hemin stimulation (n ​= ​6/group). (E) The white squares indicated perihematomal regions for microphotograph of the immunofluorescence staining. (F) Representative double immunofluorescence staining microphotograph of VDR (Green) with NeuN (Red) after ICH (n ​= ​2/group). (F) Representative double immunofluorescence staining microphotograph of VDR (Red) with NeuN (Green) after hemin stimulation (n ​= ​2/group). Data are presented as mean ​± ​SD. ∗p ​< ​0.05 vs sham group; ￡p ​< ​0.05 vs control group. Scale bar ​= ​50 ​μm.

To establish VDR as a potential therapeutic target in ICH, we first characterized its temporal and cellular expression dynamics after ICH. Western blot analysis revealed a time-dependent upregulation of VDR protein, which peaked at day 3 post-injury (Fig. 1A–C). This neuron-intrinsic response was faithfully recapitulated in vitro, where primary cortical neurons exposed to hemin exhibited a marked increase in VDR expression (Fig. 1B–D). Co-immunofluorescence staining confirmed that this upregulation was specific to the neuronal population; in the peri-hematomal cortex (Fig. 1E), VDR immunoreactivity was strongly co-localized with the neuronal marker NeuN (Fig. 1F), an observation mirrored in our in vitro model (Fig. 1G). Collectively, these findings demonstrate that VDR is specifically and dynamically upregulated in neurons subjected to hemorrhagic stress, positioning it as a key molecular responder to ICH.

### Activation of VDR attenuated neurological deficits at 1, 3, and 7 days after ICH

**Fig. 2:**
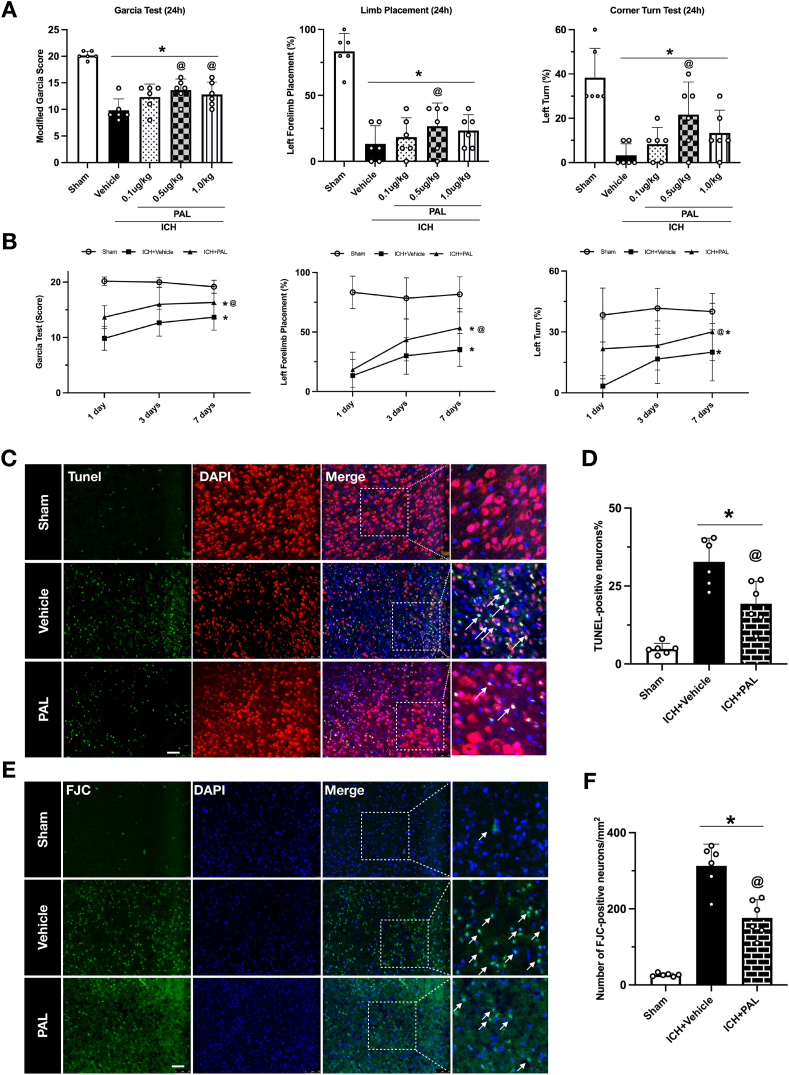
**Paricalcitol alleviates acute brain injury and neuronal death in vivo after ICH.** (A) Dose-response evaluation of paricalcitol on the modified Garcia test, forelimb placement test, and corner turn test at 24 ​h post-ICH. (B) Results of the modified Garcia test, forelimb placement test, and corner turn test at 1day, 3 days and 7days post-ICH with the optimal dose of paricalcitol. (C, D) Representative images and quantification of TUNEL staining in the peri-hematomal region at 24 ​h post-ICH. (E, F) Representative images and quantification of Fluoro-Jade C (FJC) staining in the peri-hematomal region at 24 ​h post-ICH. Data are presented as mean ​± ​SD. ∗p ​< ​0.05 vs sham group; @p ​< ​0.05 vs ICH ​+ ​vehicle group. n ​= ​6/group.

To evaluate the neuroprotective effects of VDR activation in ICH mice, paricalcitol (PAL) was administered intraperitoneally 1 ​h after ICH induction, based on prior studies and the reported maximum safe dose [28,29], three dosages (0.1 ​μg/kg, 0.5 ​μg/kg, and 1.0 ​μg/kg) were tested. Neurological function was assessed 24 ​h post-ICH using the Garcia test, Limb Placement test, and Corner Turn test. PAL treatment significantly improved neurological outcomes compared to the ICH ​+ ​Vehicle group, as evidenced by higher Garcia scores, increased left forelimb placements, and a greater percentage of left turns. Among the tested doses, 0.5 ​μg/kg demonstrated the best efficacy and was selected for subsequent evaluation (Fig. 2A).

To further assess whether the neuroprotective effect of paricalcitol was sustained, we extended our functional evaluation to days 3 and 7 post-ICH. Consistent with the 24-h results, PAL significantly enhanced neurological recovery compared to the ICH ​+ ​Vehicle group at day 3 and 7 (Fig. 2B). These results highlight that VDR activation through PAL administration effectively reduces neurological deficits in the acute phase of ICH.

### PAL inhibits ICH-induced neuronal cell death in vivo

To assess the neuroprotective effects of PAL on neuronal cell death after ICH, mice were sacrificed 24 ​h post-ICH induction. Neuronal death in the perihematomal region was evaluated using TUNEL and Fluoro-Jade C (FJC) staining. The ICH ​+ ​Vehicle group exhibited a significant increase in TUNEL-positive neurons and FJC-positive cells compared to the Sham group, indicating extensive neuronal death in the perihematomal area. PAL treatment significantly reduced both TUNEL-positive neurons and FJC-positive cells, highlighting its neuroprotective effects in vivo (Fig. 2C–F).

### Paricalcitol inhibits ferroptosis and mitochondrial damage in vivo

**Fig. 3:**
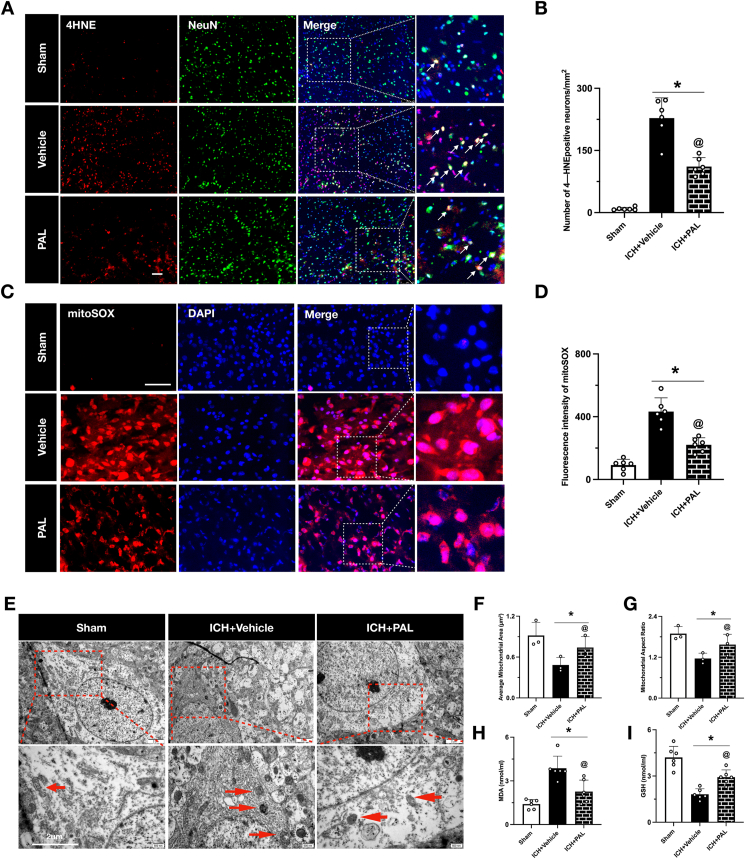
**Paricalcitol inhibits ferroptosis and mitochondrial damage in vivo.** (A, B) Representative images and quantification of double immunofluorescence staining for 4HNE and NeuN in the peri-hematomal region at 24 ​h post-ICH (n ​= ​6/group). Scale bar ​= ​200 ​μm. (C, D) Representative images and quantification of immunofluorescence staining for mitoSOX in the peri-hematomal region at 24 ​h post-ICH (n ​= ​6/group). Scale bar ​= ​200 ​μm. (E) Representative transmission electron microscopy images showing impaired mitochondria in neurons within the peri-hematomal region. Scale bar ​= ​2 ​μm. (F, G) Quantitative analysis of somatic mitochondria mean cross-sectional area and aspect ratios within the peri-hematomal region. (n ​= ​3/group). (H, I) Quantitative ELISA analysis of brain MDA and GSH level after ICH (n ​= ​6/group). Data are presented as mean ​± ​SD. ∗p ​< ​0.05 vs. sham group; @p ​< ​0.05 vs. ICH ​+ ​vehicle group.

To elucidate the mechanism of paricalcitol-mediated neuroprotection, we investigated its effect on ferroptosis and mitochondrial integrity in vivo. ICH induced robust ferroptotic stress in the peri-hematomal region, evidenced by a surge in the lipid peroxidation marker 4-Hydroxynonenal (4HNE) within neurons (Fig. 3A and B) and a marked increase in tissue levels of malondialdehyde (MDA) (Fig. 3H). This was coupled with a depletion of the endogenous antioxidant glutathione (GSH) (Fig. 3I). Paricalcitol (PAL) treatment potently suppressed ferroptosis, significantly reducing 4HNE accumulation and normalizing MDA and GSH levels.

Given the central role of mitochondria in ferroptosis, we next assessed mitochondrial-specific oxidative stress and morphology. ICH caused a dramatic increase in mitochondrial superoxide, as measured by MitoSOX fluorescence, which was markedly attenuated by PAL (Fig. 3C and D). This functional protection was mirrored at the ultrastructural level. Transmission electron microscopy revealed that while ICH induced severe mitochondrial damage—characterized by swelling, fragmentation, and cristae loss—PAL treatment preserved mitochondrial structural integrity (Fig. 3E). Quantitative morphometry confirmed that PAL prevented the ICH-induced decrease in mitochondrial aspect ratio and cross-sectional area (Fig. 3F and G). Together, these findings demonstrate that paricalcitol confers neuroprotection by directly counteracting ferroptotic signaling and preserving mitochondrial health.

### PAL-mediated inhibition of ferroptosis following ICH is independent of microglial clearance

**Fig. 4:**
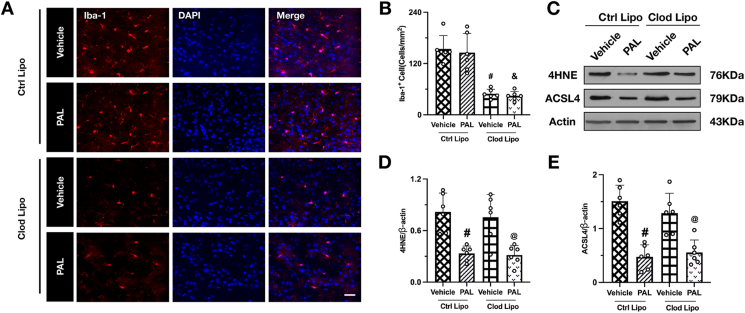
**Paricalcitol attenuates ferroptosis after ICH independently of microglial clearance.** (A) Representative immunofluorescence images of Iba-1-positive microglia (Red) in the perihematomal region after ICH. Scale bar ​= ​50 ​μm. (B) Quantitative analysis of Iba-1-positive cells in each group. (C) Representative immunoblot bands of 4HNE and ACSL4 protein levels in brain tissues from each group. (D, E) Quantitative analysis of relative protein levels for 4HNE and ACSL4. Data are presented as mean ​± ​SD. #p ​< ​0.05 vs ICH ​+ ​Ctrl Lipo ​+ ​Vehicle group; &p ​< ​0.05 vs ICH ​+ ​Ctrl Lipo ​+ ​PAL group; @p ​< ​0.05 vs ICH ​+ ​Clod Lipo ​+ ​Vehicle group. (n ​= ​6/group).

Previous work has suggested that VDR activation can promote hematoma resolution by enhancing erythrophagocytosis by mononuclear phagocytes [12]. This raises a critical alternative hypothesis: that the neuroprotective effects of paricalcitol observed in our study might be an indirect consequence of accelerated hematoma clearance, rather than a direct effect on neurons. To definitively test this possibility and isolate the neuron-intrinsic actions of PAL, we employed clodronate liposomes to deplete phagocytic microglia/macrophages in our ICH model. Immunofluorescence staining for Iba-1 confirmed the efficacy of this approach, showing a significant reduction of microglia/macrophages in the peri-hematomal region of clodronate-treated mice (Fig. 4A and B). Remarkably, the therapeutic efficacy of paricalcitol (PAL) remained intact in phagocyte-depleted animals. Western blot analysis demonstrated that PAL treatment potently suppressed the key ferroptosis markers 4-HNE and ACSL4, and this effect was not diminished by the absence of microglia/macrophages (Fig. 4C–E). This result unequivocally demonstrates that the anti-ferroptotic action of paricalcitol is not secondary to accelerated hematoma resolution but is instead a direct neuroprotective effect.

### PAL improved Long-Term neurobehavioral outcomes, reduced hippocampal CA3 neuronal degeneration, and restored dendritic spine density after ICH

**Fig. 5:**
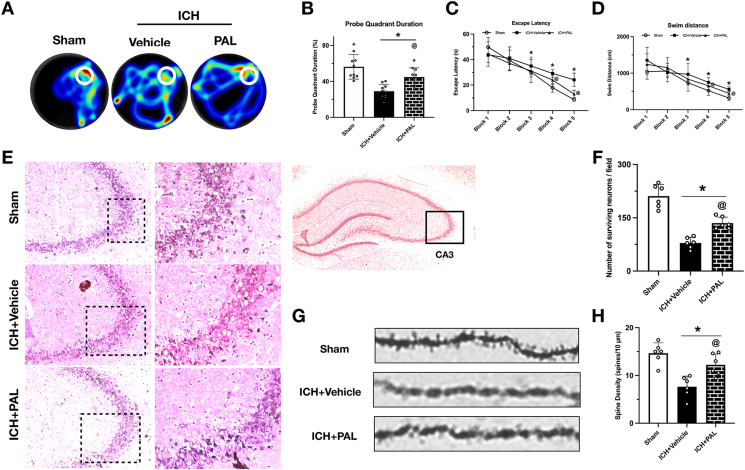
**Paricalcitol promotes long-term cognitive recovery and synaptic integrity.** (A) Heatmaps of probe trials from the Morris Water Maze. (B) Time spent in the target quadrant during the probe test on day 28 post-ICH. (C, D) Escape latency and swim distance recorded during training from days 23–27 post-ICH (n ​= ​10/group). (E) Representative Nissl staining images of the hippocampal CA3 region at 28 days post-ICH. (F) Quantification of Nissl-positive cells in the CA3 region (n ​= ​6/group). (G) Representative images with Golgi-Cox staining, (F) Quantification of dendritic spine density of neurons at 28 days after ICH. ∗p ​< ​0.05 vs. sham group; @p ​< ​0.05 vs. ICH ​+ ​vehicle group. (n ​= ​6/group).

To determine if the acute benefits of paricalcitol (PAL) translated into long-term recovery, we assessed cognitive function using the Morris water maze (MWM) at days 22–28 post-ICH. Mice in the ICH ​+ ​Vehicle group exhibited severe spatial learning and memory deficits, taking significantly longer to find the hidden platform than Sham animals. In contrast, PAL treatment conferred a remarkable improvement in cognitive performance, significantly reducing escape latencies during the acquisition phase (Fig. 5C and D). This enhanced memory was confirmed during the probe trial, where PAL-treated mice spent significantly more time in the target quadrant compared to the vehicle group, indicating superior memory retention (Fig. 5A and B).

To investigate the structural basis for this cognitive recovery, we performed histological analyses on the hippocampi of these animals. Nissl staining in the CA3 region, a critical area for spatial memory, revealed numerous darkly stained, pyknotic neurons— indicative of irreversible damage—in the ICH ​+ ​Vehicle group. PAL treatment significantly reduced the prevalence of these damaged neurons, demonstrating long-term preservation of neuronal integrity (Fig. 5E and F). To assess recovery at the synaptic level, we also performed Golgi-Cox staining. ICH induced a profound loss of dendritic spines, a key substrate for learning and memory. Remarkably, PAL treatment almost completely prevented this loss, preserving dendritic spine density at a level comparable to that of sham controls (Fig. 5G and H). Collectively, these data demonstrate that paricalcitol promotes lasting cognitive recovery by preserving both neuronal survival and synaptic integrity within the hippocampus.

### Paricalcitol confers neuroprotection via a VDR-dependent mechanism

**Fig. 6:**
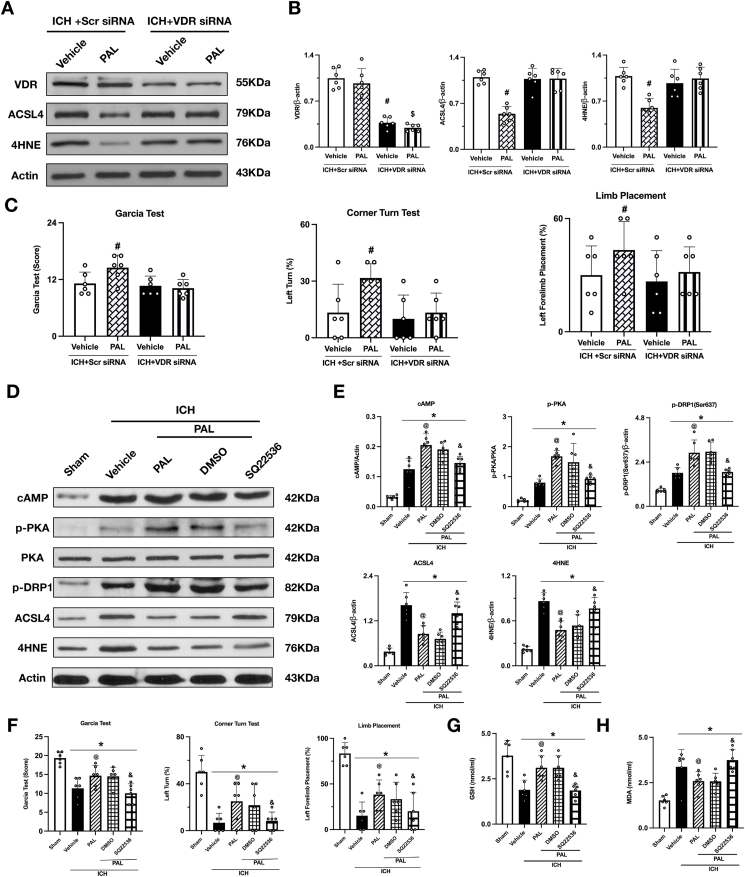
**Neuroprotection by paricalcitol in vivo is VDR-dependent and mediated by the cAMP-PKA-DRP1 pathway.** (A, B) Representative Western blot bands and quantitative analysis for VDR, ACSL4, and 4HNE after VDR siRNA administration in each group. (C) Results of the modified Garcia test, forelimb placement test, and corner turn test at 24 ​h post-ICH after VDR siRNA administration (n ​= ​6/group). (D) Representative Western blot bands for cAMP, p-PKA, PKA, p-DRP1 (Ser637), ACSL4, and 4HNE at 24 ​h post-ICH with SQ22536 injection. (E) Quantitative analysis of cAMP, p-PKA, PKA, p-DRP1 (Ser637), ACSL4, and 4HNE levels. (F) Results of the modified Garcia test, forelimb placement test, and corner turn test after SQ22536 administration. (G, H) Quantitative ELISA analysis of brain MDA and GSH level after SQ22536 administration. Data are presented as mean ​± ​SD. ∗p ​< ​0.05 vs. sham group; @p ​< ​0.05 vs. ICH ​+ ​vehicle group; &p ​< ​0.05 vs. ICH ​+ ​PAL ​+ ​DMSO group. (n ​= ​6/group). #p ​< ​0.05 vs ICH ​+ ​Vehicle ​+ ​Scr siRNA group; $p ​< ​0.05 vs ICH ​+ ​PAL ​+ ​Scr siRNA group; ∗p ​< ​0.05 vs. sham group; @p ​< ​0.05 vs. ICH ​+ ​vehicle group; &p ​< ​0.05 vs. ICH ​+ ​PAL ​+ ​DMSO group. (n ​= ​6/group).

To definitively test if the neuroprotective effects of paricalcitol are VDR-dependent, we employed an siRNA-mediated knockdown strategy in vivo. Following confirmation of efficient VDR protein knockdown (Fig. 6A and B), we assessed both molecular and functional outcomes. In control mice receiving scrambled siRNA, paricalcitol potently suppressed the key ferroptosis markers ACSL4 and 4HNE and significantly improved neurological scores. Crucially, these therapeutic benefits were completely abolished by VDR knockdown. In the absence of VDR, paricalcitol was rendered ineffective: it failed to reduce ACSL4 and 4HNE levels, and the corresponding functional recovery was lost (Fig. 6A–C). These results provide conclusive evidence that VDR is indispensable for the anti-ferroptotic and neuroprotective actions of paricalcitol.

### The VDR-dependent effects of paricalcitol are mediated by the cAMP-PKA-Drp1 pathway

Having established that paricalcitol's neuroprotective action is VDR-dependent, we next sought to delineate the downstream signaling cascade. We hypothesized that VDR activation engages the cAMP-PKA pathway to regulate Drp1, a key mediator of mitochondrial dynamics and ferroptosis. To test this, we performed a pharmacological loss-of-function experiment using the adenylyl cyclase inhibitor SQ22536. As hypothesized, inhibiting the cAMP pathway completely nullified the therapeutic efficacy of PAL. Co-administration of SQ22536 not only prevented the PAL-induced phosphorylation of PKA and Drp1 (Ser637) but also completely reversed its downstream anti-ferroptotic effects. Specifically, SQ22536 abrogated PAL's ability to suppress the key ferroptosis drivers ACSL4 and 4HNE, normalize lipid peroxidation (MDA levels), and restore the endogenous antioxidant glutathione (GSH). This molecular failure translated directly to a functional one, as SQ22536 concurrently abolished the neurological recovery promoted by PAL (Fig. 6D–H). These results demonstrate that the cAMP-PKA-Drp1 pathway is the essential downstream transducer of PAL's VDR-dependent, anti-ferroptotic action.

To provide complementary, gain-of-function evidence, we used Forskolin (FSK) to directly activate adenylyl cyclase. As hypothesized, FSK treatment alone mimicked the key molecular effects of PAL, significantly increasing PKA phosphorylation and suppressing Drp1 phosphorylation (Ser616), which culminated in the downregulation of the ferroptosis markers ACSL4 and 4HNE (Figure S1A-E). The convergent findings from this dual pharmacological approach—whereby inhibiting the pathway blocks PAL's effects and activating it mimics them—provide compelling evidence that the cAMP-PKA-Drp1 axis is the critical downstream mediator of paricalcitol-induced neuroprotection.

### Paricalcitol protects neurons from hemin-induced death in vitro via a VDR- and cAMP-dependent mechanism

**Fig. 7:**
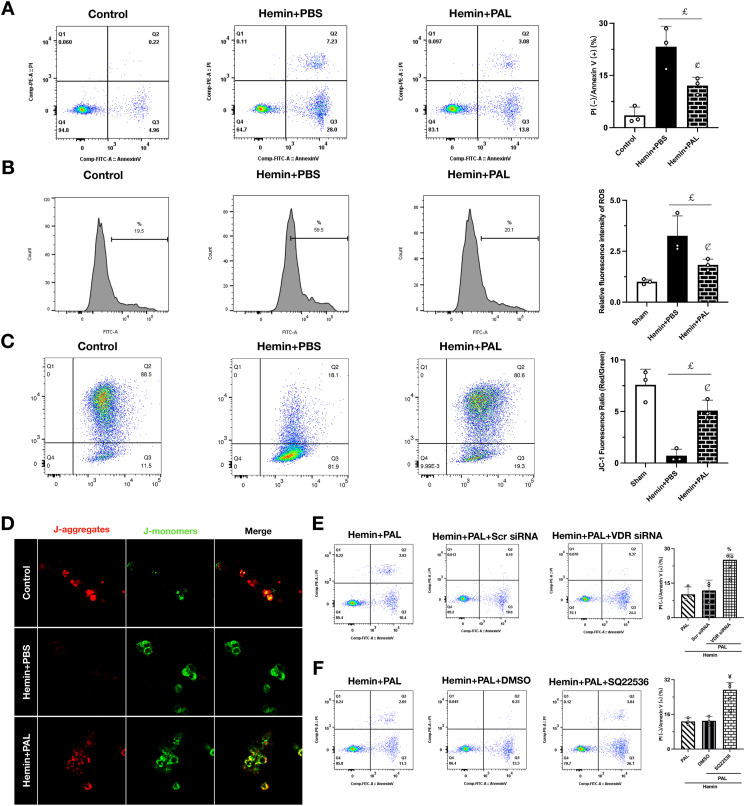
**Paricalcitol protects neurons from hemin-induced death in vitro via a VDR- and cAMP-dependent mechanism.** (A) Flow cytometry dot plots and quantification of neuronal survival and apoptosis. PI (−)/Annexin V (+) indicates early apoptotic neurons. (B) Flow cytometry dot plots and quantification of ROS levels after hemin stimulation. (C) Flow cytometry dot plots and quantification of JC-1 staining for mitochondrial membrane potential. (D) Representative immunofluorescence images of JC-1 staining after hemin stimulation. (E) Flow cytometry dot plots and quantification of neuronal survival and apoptosis after VDR knockdown. PI (−)/Annexin V (+) indicates early apoptotic neurons. (F) Flow cytometry dot plots and quantification of neuronal survival and apoptosis after SQ22536 administration. PI (−)/Annexin V (+) indicates early apoptotic neurons. %p ​< ​0.05 vs. Hemin ​+ ​Scr siRNA group. ¥p ​< ​0.05 vs. Hemin ​+ ​PAL ​+ ​DMSO group.

To corroborate our in vivo findings at the cellular level, we established an in vitro model of hemorrhagic injury using primary neurons exposed to hemin. Flow cytometry analysis revealed that hemin treatment induced significant neuronal apoptosis, as measured by Annexin V/PI staining. Paricalcitol (PAL) treatment robustly counteracted this, significantly preserving neuronal viability and reducing the apoptotic population (Fig. 7A). This neuroprotective effect was linked to the mitigation of oxidative stress and the preservation of mitochondrial health. PAL effectively reversed the hemin-induced surge in intracellular ROS (Fig. 7B) and prevented the collapse of the mitochondrial membrane potential, a key indicator of mitochondrial dysfunction (Fig. 7C). This was confirmed by both JC-1 flow cytometry and, critically, by in situ JC-1 immunofluorescence on intact neurons (Fig. 7D).

Finally, we used this in vitro system to definitively test the proposed signaling pathway. As hypothesized, the anti-apoptotic benefit of PAL was completely VDR-dependent, as siRNA-mediated knockdown of VDR completely abrogated its protective effect (Fig. 7E). Furthermore, the downstream involvement of the cAMP-PKA cascade was confirmed, as the adenylyl cyclase inhibitor SQ22536 also fully reversed the neuroprotection conferred by PAL (Fig. 7F). Taken together, these in vitro results provide direct evidence that paricalcitol protects neurons from hemorrhagic stress by preserving mitochondrial integrity through a mechanism that requires both VDR and the cAMP-PKA signaling pathway.

## Discussion

The neuroprotective potential of VDR activation is increasingly evident across neurological disorders. However, its role in modulating mitochondrial dynamics and ferroptosis during ICH remains poorly understood. In this study, we demonstrated that activating VDR with paricalcitol significantly reduces neuronal ferroptosis after ICH. Specifically, VDR activation inhibited excessive mitochondrial fission through the cAMP-PKA-DRP1 signaling pathway, thereby preserving mitochondrial integrity and functionality. This process ultimately mitigated neuronal death and improved neurological outcomes in ICH. These findings highlight the critical role of VDR in ferroptosis regulation and provide a promising therapeutic strategy for improving post-ICH recovery.

VDR, a nuclear receptor regulating calcium homeostasis and mitochondrial dynamics [35,36], exhibits neuroprotective protein expression changes localized to stressed neurons across neurological disorders. In ischemic stroke, VDR levels surge in peri-infarct neurons within 72 ​h post-injury, mitigating oxidative damage [29,37]. Similarly, hippocampal and cortical neurons in Alzheimer's and Parkinson's models show upregulated VDR correlated with neuronal survival [38,39]. It is well-established that VDR is also expressed in glial cells, including astrocytes and microglia, under various conditions [40,41]. However, in our acute ICH model, we observed that VDR expression was predominantly localized to neurons. Our study also identifies a conserved spatiotemporal pattern in ICH: VDR expression peaked three days post-injury in peri-hematoma neurons, a neuron-specific response replicated in vitro under hemin-induced hemorrhagic stress. This aligns with VDR's neuroprotective colocalization in peri-infarct neurons [42] and hippocampal neurons [43] from other pathologies. Notably, ICH-driven upregulation shares mechanistic triggers with ischemia and neurodegeneration—iron toxicity, ROS, and neuroinflammation [44,45]—all hallmarks of hemorrhagic injury. For example, hemin-mediated iron overload in our model directly induced neuronal VDR expression, mirroring the ROS-dependent upregulation reported in ischemic neurons [29]. Such parallels establish neuronal VDR as a spatially (peri-injury neurons) and temporally (3-day peak) calibrated adaptive responder. Our findings thus unify VDR's dynamics across neurological disorders while highlighting its therapeutic potential to enhance endogenous neuroprotection after ICH. The primary endogenous ligand for VDR in the brain is 1,25-dihydroxyvitamin D3 (1,25(OH)2D3), which mediates its functions through the classic nuclear receptor signaling pathway [46]. The brain possesses the capability for local synthesis and degradation of 1,25(OH)2D3, endowing the central nervous system with an independent, finely regulated vitamin D signaling system essential for maintaining neural homeostasis [47].

VDR has been extensively studied for its neuroprotective roles across various neurological disorders, including spinal cord injury, neurodegenerative diseases such as Alzheimer's and Parkinson's, and ischemic stroke [[48], [49], [50], [51], [52], [53]]. In these models, VDR activation has been shown to reduce neuronal apoptosis, promote axonal regeneration, alleviate functional deficits, mitigate oxidative stress, suppress neuroinflammation, and enhance neuronal survival. Building on these insights, our study is the first to demonstrate the neuroprotective effects of VDR activation in an ICH model. Using paricalcitol, a selective VDR agonist, we found that VDR activation significantly reduced neuronal damage and improved both early and long-term neurological outcomes, suggesting that VDR activation through paricalcitol can mitigate neuronal injury and promote functional recovery in the acute and chronic phases of ICH. Paricalcitol was selected as the therapeutic agent due to its superior pharmacological properties and clinical potential. Compared to other VDR agonists such as calcitriol, paricalcitol offers enhanced safety by significantly reducing the risk of hypercalcemia, a major side effect associated with VDR activation [54]. This improved safety profile makes paricalcitol more suitable for long-term therapeutic use, particularly in conditions like ICH that may require prolonged intervention. Additionally, paricalcitol has demonstrated neuroprotective effects in preclinical studies of central nervous system disorders and can cross the blood-brain barrier, enabling it to target brain tissues directly and exert its protective effects [55]. Mitochondrial dysfunction and ferroptosis are pivotal pathological mechanisms in the development of various neurological and systemic diseases, and VDR has been identified as a critical regulator in these processes. Studies have established that VDR activation bolsters mitochondrial function by modulating oxidative phosphorylation, curbing mitochondrial fragmentation, and alleviating oxidative stress [56,57]. Additionally, VDR has been shown to suppress ferroptosis through the regulation of antioxidant defenses, notably by influencing the expression of glutathione peroxidase 4 (GPX4), a central enzyme in the inhibition of ferroptosis [58,59]. In our study, we demonstrated that VDR activation significantly mitigates mitochondrial fission, reduces ROS production, and inhibits neuronal ferroptosis, leading to a decrease in neuronal death and an improvement in neurological outcomes. These findings suggest that the neuroprotective effects of VDR activation may be mediated through the suppression of mitochondrial dynamics-related ferroptosis, presenting a novel and promising avenue for ICH research.

Our findings along with previous studies, have established that VDR plays a crucial role in regulating mitochondrial function and ferroptosis. However, the specific molecular mechanisms remain unclear. Emerging evidence suggests that VDR may influence cellular signaling pathways critical for stress response and mitochondrial regulation [56]. In models of diabetic nephropathy, VDR activation has been shown to upregulate adenylyl cyclase expression, leading to increased intracellular cAMP levels and enhanced cellular stress responses [60,61]. In the Helicobacter pylori infection model, it was discovered that activating the VDR can upregulate the expression of cAMP[62]. cAMP, as a second messenger, activates PKA by binding to its regulatory subunits, causing the release of its catalytic subunits. Activated PKA subsequently phosphorylates downstream targets, including DRP1 at the Ser637 site. This phosphorylation inhibits DRP1's pro-fission activity, stabilizing mitochondrial dynamics and preserving energy homeostasis [[63], [64], [65]]. In the nervous system, studies in Parkinson's disease models have demonstrated that PKA-mediated DRP1 phosphorylation reduces mitochondrial fragmentation, protects dopaminergic neurons, and alleviates motor deficits [66,67]. Similarly, in traumatic brain injury, PKA activation was shown to prevent DRP1-mediated mitochondrial fission, reducing neuronal apoptosis and improving functional recovery [68]. These findings underscore the pivotal role of the cAMP-PKA axis in regulating DRP1 activity and maintaining mitochondrial integrity in the brain. In our study, VDR activation by paricalcitol significantly enhanced cAMP levels, activated PKA, and promoted DRP1 phosphorylation at the Ser637 site. These molecular changes were associated with reduced mitochondrial fission, improved mitochondrial function, and decreased neuronal ferroptosis in both in vivo ICH models and in vitro experiments. Importantly, the protective effects of VDR activation were abolished by either siRNA-mediated VDR knockdown or the cAMP inhibitor SQ22536. Inhibition of cAMP signaling disrupted PKA activation and reduced DRP1 phosphorylation, indicating that the cAMP-PKA signaling axis is a critical mediator of the observed mitochondrial protection. These findings suggest that VDR exerts its neuroprotective effects, at least in part, through the cAMP-PKA-DRP1 signaling pathway, thereby modulating mitochondrial dynamics and attenuating ferroptosis under pathological conditions.

There are several limitations in this study. First, we used siRNA-mediated VDR knockdown and the cAMP inhibitor SQ22536 to investigate the role of the VDR-cAMP-PKA-DRP1 signaling pathway in our study. However, genetic animal models with VDR knockout or DRP1 phosphorylation site-specific mutations are needed to further validate our findings. Second, we primarily focused on the cAMP-PKA-DRP1 signaling pathway as the underlying mechanism of VDR-mediated neuroprotection in ICH. Other molecular signaling pathways that may contribute to the observed effects, such as the PI3K-Akt or MAPK pathways, were not explored. Lastly, this study did not evaluate potential sex differences in the neuroprotective effects of VDR activation. Considering that hormonal differences may influence ferroptosis and mitochondrial function, future studies should include both male and female animals to provide more comprehensive insights.

## Conclusion

VDR plays a critical role in protecting mitochondrial function and preventing ferroptosis in ICH. Activation of VDR by paricalcitol attenuated mitochondrial dysfunction, reduced neuronal ferroptosis, and improved neurobehavioral outcomes through the cAMP-PKA-DRP1 signaling pathway in ICH mouse models in vivo and hemoglobin-stimulated primary neuron cultures in vitro. Thus, VDR activation may serve as a potential therapeutic strategy for alleviating neuronal damage and improving recovery after ICH.

## Ethical Approval and Consent to Participate

All animal procedures were approved by the Institutional Animal Care and Use Committee (IACUC) of the First Affiliated Hospital of the University of Science and Technology of China (2022-N(A)-300) and conducted in compliance with ethical guidelines.

## Consent for publication

Not applicable.

## Data availability statement

The data support the findings of this study and are available from the corresponding author upon reasonable request.

## Author contributions

Mi Tian: Writing – original draft, Software, Methodology, investigation, Formal analysis, Data curation, Conceptualization. Xin Li: Methodology, investigation, Formal analysis, Data curation. Dongqing Qi: Writing – original draft, Software, Formal analysis, Data curation, Conceptualization. Pengju Wei: review & editing, Supervision. Peng Jin: original draft, Software, Methodology, investigation, Formal analysis, Data curation, Conceptualization, Supervision and Funding acquisition.

## Funding

This study was supported by grants from 10.13039/501100003995Anhui Provincial Natural Science Foundation (2208085MH234 to Peng Jin).

## Declaration of competing interest

The authors declare that they have no known competing financial interests or personal relationships that could have appeared to influence the work reported in this paper.
