# Supplementary material for: Paricalcitol-mediated vitamin D receptor activation attenuates neuronal ferroptosis via cAMP-PKA-DRP1 signaling pathway after intracerebral hemorrhage
Source: Neurotherapeutics. 2025 Oct 21;23(1):e00767. doi: 10.1016/j.neurot.2025.e00767 (PMC12976504; doi:10.1016/j.neurot.2025.e00767)
Supplement: Multimedia component 1 [file mmc1.docx]

| Table S1. Summary of experimental groups and mortality rate in the study. | | | | | | | | |  |
| --- | --- | --- | --- | --- | --- | --- | --- | --- | --- |
|  |  |  |  |  |  |  |  |  |  |
| **Experimental Groups** | | **Neurological test** | **IHC** | **MDA/**  **GSH** | **WB** | **Exclusion** | **Mortality (%)** | **Subtotal** | |
| **Experimental 1** | |  |  |  |  |  |  |  | |
| Sham | |  | 2 |  | 6 | 0 | 0 | 8 | |
| ICH (6h, 12h, 24h, 72h, 7d) | |  | 2 |  | 30 | 1 | 1 (2.94%) | 34 | |
| **Experimental 2** | |  |  |  |  |  |  |  | |
| Sham (24h) | | 6 |  |  |  | 0 | 0 | 6 | |
| ICH + Vehicle (24h) | | 6 |  |  |  | 0 | 1 (14.29%) | 7 | |
| ICH + PAL 0.1ug//kg (24h) | | 6 |  |  |  | 0 | 0 | 6 | |
| ICH + PAL 0.5ug//kg (24h) | | 6 |  |  |  | 0 | 0 | 6 | |
| ICH + PAL 1.0ug//kg (24h) | | 6 |  |  |  | 0 | 1 (14.29%) | 7 | |
| Sham (72h, 7d) | | 6 |  |  |  |  |  | 6 | |
| ICH + Vehicle (72h, 7d) | | 6 |  |  |  |  |  | 6 | |
| ICH + PAL 0.5ug//kg (72h, 7d) | | 6 |  |  |  | 0 |  | 6 | |
| **Experimental 3** | |  |  |  |  |  |  |  | |
| Sham | | 10 | 0 | 6 | 0 | 0 | 0 | 16 | |
| ICH + Vehicle | | 10 | 0 | 6 | 0 | 0 | 1 (6.25%) | 16 | |
| ICH + PAL | | 10 | 0 | 6 | 0 | 0 | 1 (6.25%) | 16 | |
| **Experimental 4** | |  |  |  |  |  |  |  | |
| ICH + Control Liposomes + Vehicle | | 6 |  |  |  |  |  | 6 | |
| ICH + Control Liposomes + PAL | | 6 |  |  |  |  |  | 6 | |
| ICH + Clodronate Liposomes + Vehicle | | 6 |  |  |  |  |  | 6 | |
| ICH + Clodronate Liposomes + PAL | | 6 |  |  |  |  |  | 6 | |
| **Experimental 5** | |  |  |  |  |  |  |  | |
| ICH +Vehicle+Scr siRNA | | 6 |  | 6 |  | 0 | 0 | 12 | |
| ICH+Vehicle+VDR siRNA | | 6 |  | 6 |  | 0 | 0 | 12 | |
| ICH+ PAL+Scr siRNA | | 6 |  | 6 |  | 0 | 1 (7.69%) | 13 | |
| ICH+ PaL+VDR siRNA | | 6 |  | 6 |  | 0 | 1 (7.69%) | 13 | |
| Sham | |  |  |  | 6 |  |  | 6 | |
| ICH+Vehicle | |  |  |  | 6 |  |  | 6 | |
| ICH+PAL | |  |  |  | 6 |  |  | 6 | |
| ICH+PAL+DMSO | |  |  | 6 | 6 |  | 1 (7.69%) | 13 | |
| ICH+PAL+SQ22536 | |  |  | 6 | 6 |  | 1 7.69%) | 13 | |
| **Total** | |  |  |  |  |  | 9 (3.46%) | 260 | |
|  | ICH, intracerebral hemorrhage; WB, western blot; IF: Immunofluorescence; DMSO, dimethyl sulfoxide | | | | | | | | |


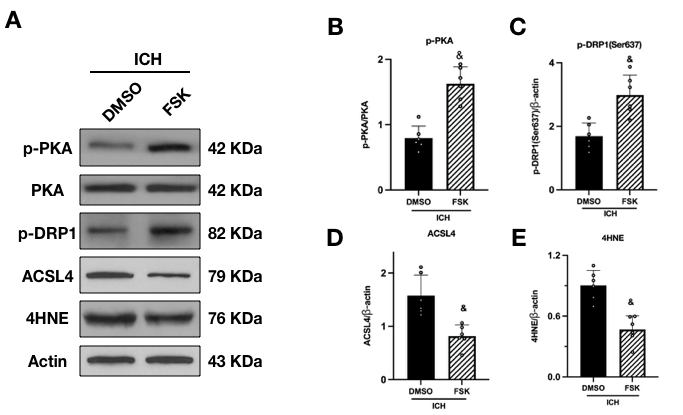


**Figure S1. Direct activation of the cAMP-PKA pathway with Forskolin mimics the anti-ferroptotic molecular changes induced by Paricalcitol in vivo.** (A) Representative Western blot bands for p-PKA, PKA, p-DRP1 (Ser637), ACSL4, and 4HNE at 24 hours post-ICH. (B) Quantitative analysis of p-PKA, PKA, p-DRP1 (Ser637), ACSL4, and 4HNE levels. Data are presented as mean ± SD (n = 6 per group). &p < 0.05 vs. the ICH+DMSO group.
